# Introductory Lectures

**Published:** 1849-02

**Authors:** 


### INTRODUCTORY LECTURES.

We continue our notice of the Introductory Lectures which have been politely sent to us.

Dr. George W. Norris, Professor of Clinical Surgery in the University of Pennsylvania, delivered his first introductory lecture to the class at the Pennsylvania Hospital, on the first of November. It is such a production as those who know Dr. Norris would expect from him—a sensible, practical discourse, written in a plain, unambitious style, and instructive upon those points which students are particularly interested in understanding before they enter upon the duties of their profession. "Students" he says, "are very apt to take up the false notion, that it is by operations that a great and lasting surgical reputation is to be attained. Nothing," he adds, "is further from the truth." And he adduces examples showing how surgery has advanced in this respect, and how bloody operations once deemed indispensable, one after another, have been superseded.

"Ulcers and diseased joints, classes of diseases in which amputation was formerly so common, are now by improved methods of treatment in the great majority of cases cured without resort to operative means. Castration, which in former days was so common an operation in chronic diseases of the testis, is now rarely done—so rarely, that in this hospital, with which I have been connected for fifteen years, and in which many such affections are treated, I have never seen it once performed here. The application of the trephine, too, at one period so frequent, is now rarely resorted to, and within a very short time, the treatment of aneurisms by pressure has been so improved upon by an accurate study of the process employed by nature in her spontaneous cures of that disease, and such an adaptation of the treatment as to imitate her, that there is good reason to hope that in the extremities at least, another bloody operation will be henceforth in many cases deemed unnecessary."

The lecture of Dr. Merrick, Professor of Chemistry and Botany in the Starling Medical College, at Columbus, O., treats of the pursuit of knowledge. It evinces in its author thought, cultivation, and poetry. The style in which it is written is pleasing, and the illustrations from the natural world, from biography and history, are apposite, and convey a useful lesson. We do not admire the quotation from Mr. Tupper. Dr. Merrick could have expressed the same thoughts quite as forcibly, and much less affectedly, in his own language.

The lecture of Dr. George R. Grant, Professor of the Theory and Practice of Medicine in the Memphis Medical College, is in a more ambitious style. His subject is the importance of professional studies, and he treats it with earnestness and ability, drawing from the lives of the eminent men of our profession examples that must have made a salutary impression upon his pupils. If we excepted to Dr. Grant's interesting lecture in any respect, it would be that he quotes more poetry than the subject seems to us to demand. Mr. Tupper appears to be a great favorite with medical professors. Dr. Grant too has a quotation from his "Proverbial Philosophy." The thought is a good one, but was much better expressed, long ago, by Solomon in his proverb, *"train up a child in the way he should go,"* &c.

*A Discourse on the Influence of Diseases on the Intellectual and Moral Powers.* This is the title of the introductory lecture of Dr. Joseph Mather Smith, of the College of Physicians and Surgeons in the City of New York. The discourse is more elaborate than we generally find the lectures delivered on such occasions, and yet treats the interesting subject in a way to render it instructive to a popular audience. Prof. Smith does not appear to be a very decided phrenologist; or at least he did not feel bound to decide the question "whether the cerebral apparatus is a single organ, having an individuality of function, or a congeries or plurality of organs, each having a special function." He treats his subject rather metaphysically than phrenologically—rather after the fashion of Stewart and Abercrombie, than of Spurzheim and Combe. The lecture reminds us of those of our great countryman, Rush, delivered on kindred subjects, and which we read long ago with all the pleasure that we found in books of poetry and romance. The lecture of Dr. Smith possesses more than an ephemeral interest, and will repay a careful perusal.

